# Effect of Hip Flexion Angle on the Hamstring to Quadriceps Strength Ratio

**DOI:** 10.3390/sports7020043

**Published:** 2019-02-15

**Authors:** Eleftherios Kellis, Athanasios Ellinoudis, Nikolaos Kofotolis

**Affiliations:** Laboratory of Neuromechanics, Department of Physical Education and Sport Science at Serres, Aristotle University of Thessaloniki, Thessaloniki 54124, Greece; ellinoud@phed-sr.auth.gr (A.E.); kofotol@phed-sr.auth.gr (N.K.)

**Keywords:** isokinetics, hamstring injury, muscle length, strength imbalance, hamstring strain, rehabilitation

## Abstract

The purpose of this study was to compare the hamstring to quadriceps ratio (H:Q) obtained from three different hip flexion angles. Seventy-three young athletes performed maximum isokinetic concentric and eccentric knee extension and flexion efforts at 60 °·s^−1^ and 240 °·s^−1^ from hip flexion angles of 90°, 60°, and 120°. The conventional (concentric to concentric), functional (eccentric to concentric) and mixed (eccentric at 30 °·s^−1^ to concentric torque at 240 °·s^−1^) H:Q torque ratios and the electromyographic activity from the rectus femoris and biceps femoris were analyzed. The conventional H:Q ratios and the functional H:Q ratios at 60 °·s^−1^ did not significantly differ between the three testing positions (*p* > 0.05). In contrast, testing from the 90° hip flexion angle showed a greater functional torque ratio at 240 °·s^−1^ and a mixed H:Q torque ratio compared with the other two positions (*p* < 0.05). The hip flexion angle did not influence the recorded muscle activation signals (*p* > 0.05). For the range of hip flexion angles tested, routine isokinetic assessment of conventional H:Q ratio and functional H:Q ratio at slow speed is not angle-dependent. Should assessment of the functional H:Q ratio at fast angular velocity or the mixed ratio is required, then selection of hip flexion angle is important.

## 1. Introduction

Hamstring muscle strains and knee joint injuries are very common in soccer [1] with a very high recurrence rate [2]. Hamstring muscle strength weakness is frequently considered an injury risk factor in soccer players [3]. There are various indices of hamstring muscle strength which have been associated with injury risk, such as maximum muscle strength, the bilateral limb strength ratio and the hamstrings (H) to quadriceps (Q) muscle strength ratio [4,5,6,7,8,9]. In most cases, these strength indices are routinely assessed using isokinetic dynamometers [4,5,6,7,8,9,10,11,12]. 

The H:Q muscle strength ratio is frequently considered an indicator of strength balance of antagonistic muscle groups around the knee joint [4,5,6,7,8,9]. Traditionally, the conventional ratio is estimated by dividing the concentric H (H_CON_) by the concentric Q (Q_CON_) or the eccentric H (H_ECC_) by the eccentric Q (Q_ECC_) torque ratio [13]. The functional or dynamic control (H_ECC_/Q_CON_) torque ratio is based on the concept that during functional movements, the agonist muscle shortens while the antagonist muscle lengthens [10,14]. More recently, a new version of the functional ratio, the ‘mixed’ ratio, defined as the H_ECC_ torque at an angular velocity of 30 °·s^−1^ divided by the Q_CON_ torque at 240 °·s^−1^ was also proposed [5,11]. This ratio was proposed by Croiser et al. [5,11] for two reasons: first, because it resembles the function of the muscles during sprinting and kicking and, second, because isokinetic eccentric testing at a slow angular velocity allows a more valid assessment of the peak H torque compared with high angular velocity tests [5,11].

Research findings on the association between an altered H/Q ratio and injury risk appear conflicting as some studies have reported that the H:Q ratio has no association with hamstring injury [7,8] while others have reported the opposite [5,15,16,17]. Recently, Dauty et al. [6] found that a conventional ratio less than 0.47 obtained at an isokinetic velocity of 180 °·s^−1^ had similar or better predictive value compared with the mixed ratio. However, latest research in soccer players indicated that the use of H:Q ratio as an injury predictor largely depends on the cut-off value [18]. This conflicting evidence indicates that further examination of strength imbalances might be necessary.

Researchers have been commented that the assessment of H:Q ratio with the hip flexion angle of 90° (standard seated position of the isokinetic dynamometer) may not resemble kinematics of the late phase of the sprinting cycle where the athlete flexes the hip by 75–90° and the knee flexion angle is 30° [19]. For the same reason, strength evaluation from the standard seated position may not be reflect strength imbalances that occur during the early contact phase, where the hip is in neutral position and the knee is almost extended [20,21]. An increase in hip flexion angle has opposing effects on the length of the antagonist muscle groups by increasing the length of the bi-articular hamstrings and decreasing the length of the bi-articular quadriceps. Studies have shown that an increase in hip flexion angle is accompanied by a greater peak torque of the knee flexors [22,23,24] while knee extensor torque was reported either to increase [25,26] or to be unaffected by hip flexion angle [23]. Research studies have shown that an increase in hip flexion angle from 0° to 90° increases the H:Q ratio [20,23,25]. It was proposed that evaluation of the isokinetic H:Q ratio with a hip flexion angle of 10° [20] or 80° [23] is ecologically more valid [20] than the standard seated position.

Studies have reported that athletes with a previous hamstring injury display a shift of peak torque towards greater knee flexion angles (shorter lengths) [27]. This finding led some investigators to incorporate hamstring exercises at longer muscle lengths [28,29]. In one of those studies, the isokinetic dynamometer was modified to allow exercising from a greater hip flexion angle (>120°) to better mimic exercise injury conditions [29]. This raises the question whether isokinetic evaluation of the H:Q ratio at hip flexion angles that are greater than the standard seated position may yield different results. Furthermore, while previous studies have reported that hip flexion angle has limited influence on muscle activation patterns [22,23,30], it is not clear whether this applies to hip flexion angles that are greater than 90°.

Although the effects of hip flexion angle on peak knee extension and flexion torque and conventional ratios have been investigated [20,22,23,25], a few reports focused on the evaluation of the functional H:Q ratio [20,23]. Further, no previous study has examined the influence of hip flexion angle on the mixed H:Q ratio while H:Q ratios with the hip flexion angle greater than 120° have not been previously published. There is evidence that the alteration of hip flexion angle has greater effect on eccentric than concentric torque and this effect may vary between the two antagonistic muscle groups [20]. Consequently, this may indicate that changing hip flexion angle may not have the same influence on the conventional, functional, and mixed H:Q ratios.

There are various factors that may influence the exerted torque around a joint, including, muscle length, activation, architecture, and moment-arm. Changes in muscle length of the quadriceps and hamstrings as a result of changes in the hip and knee joint angles may also influence their respective level of activation. Deighan et al. [20] hypothesized that the effects of hip flexion angle on H:Q torque ratio may be due to an altered neuromuscular activation. Nevertheless, no electromyographic (EMG) data were collected to support this argument. Guex et al. [23] reported that although a greater hip flexion angle increased the H:Q ratios, there were no changes in hamstring EMG patterns. However, no EMG data from the quadriceps was obtained. A recent study [31] has reported that changing hip flexion angle had no influence on hamstring and quadriceps EMG, but these data refer only to concentric isokinetic measurements while no H:Q ratios were calculated. Simultaneous examination of both muscle activation may provide a deeper understanding of the influence of hip flexion angle on the exerted torque, and, hence, the H:Q ratio.

Although muscle strength and strength balance between antagonistic muscle groups are recognized as potential risk factors for soccer injuries, a recent review of the literature has concluded that the evidence regarding the capacity of such indices to predict injury is inconclusive [3]. This indicates that the development of isokinetic testing protocols that take into consideration the mechanism of injury and the corresponding function of the antagonistic muscle groups is necessary. The aim of this study was to examine the effect of three different hip flexion angles on the conventional, functional, and mixed H:Q torque ratios. Based on the previous literature, three hypotheses were tested: first, that the hip flexion angle would affect the isokinetic conventional H:Q ratio, second, that the hip flexion angle would alter the functional and the mixed H:Q ratios and, third, that the hip flexion angle would have no effect on the muscle activation of quadriceps and hamstrings.

## 2. Materials and Methods

### 2.1. Subjects

A total 73 healthy male volunteers (age 22.71 ± 0.35 years; height 176.4 ± 4.3 cm; body mass 80.3 ± 4.4 kg) took part in this investigation. To be included in the study, the athletes should have no musculoskeletal injury for the past year prior to the measurement session. Participants answered a health questionnaire and signed a written informed consent document. All participants were amateur athletes engaging in sports (soccer, basketball, track-and-field, handball, tennis, and volleyball) training four times a week. The protocol was approved by the University’s institutional research ethics committee.

### 2.2. Procedures

**Figure 1:**
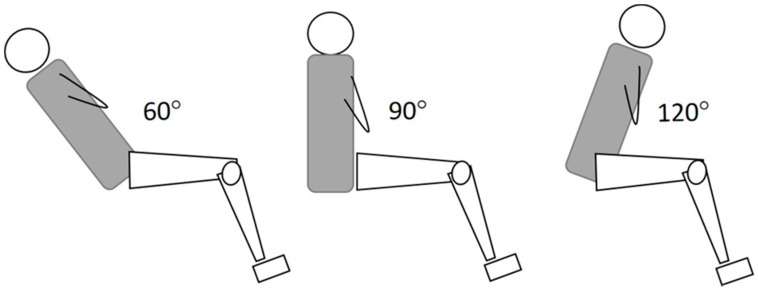
Illustration of the three positions. Testing was performed in the standard seated position (hip flexion angle = 90°), a greater (120°) and a lower hip flexion angle (60°).

For the assessment of maximum strength, a Humac Norm isokinetic dynamometer (Cybex CSMI, Stoughton, MA, U.S.A.) was used. The main protocol included performance of maximum strength efforts of the knee flexors and extensors of the right (preferred) limb from the seated position with three different hip flexion angular positions (Figure 1): hip flexion angle of 90° (H90), as recommended by the manufacturer, hip flexion angle of 120° (H120) and, third, hip flexion angle of 60° (H60). A standard analogue goniometer was used to monitor the hip joint angle (accuracy of ±1°) at rest and during a maximum voluntary contraction (MVC). Velcro straps were used to stabilize the trunk, pelvis, and thigh. The dynamometer axis of rotation and the knee joint rotation (at the level of lateral femoral condyle) were aligned. To enable better alignment of the two axes additional lifting and rotation of the dynamometer main unit was required in the H60 position. The limb and dynamometer input arm were then weighed by holding the knee statically at a knee flexion angle of 30°, as recommended by the manufacturer. The range of motion of the knee joint was set from 0° (full extension) to 90° of knee flexion. 

Each participant was familiarized with the testing protocol by performing five submaximal efforts of the knee extensors and flexors at 30 °·s^−1^ from each of the three hip flexion angles. This was followed by performance of efforts of the knee extensors and flexors as follows from three hip flexion angles: one set of five repetitions at a concentric angular velocity of 30 °·s^−1^, one set of five repetitions at a concentric angular velocity of 240 °·s^−1^, one set of five repetitions at an eccentric angular velocity of 30 °·s^−1^, and one set of five repetitions at an eccentric angular velocity of 240 °·s^−1^. The subjects received instructions to exert maximum force as fast as possible throughout the whole trial. The time interval between sets was 1 min to reduce the potential influence of fatigue. The order of testing was randomized between testing positions, mode of testing (eccentric, concentric) and angular velocities. 

In addition, the maximum isometric contraction (MVC) force was evaluated by performing three efforts from a hip flexion angle of 90° and a knee angle at 30° (for knee flexors) and 65° (for knee extensors). Selection of these angles was based on the observation that they display the greater torque during isokinetic knee extension—flexion tests. 

Of the five isokinetic trials, the trial which displayed the greater gravity-corrected torque was used for further analysis. To reduce the effects of acceleration on isokinetic data, the first and last 10° in the range of motion were excluded from the analysis. From the raw torque–angular position curve of the selected repetition, the peak torque value was identified and used for further analysis. In each hip testing position, we calculated: (a) the conventional ratios using concentric torques at 30 °·s^−1^ (H_CON30_/Q_CON30_) and 240 °·s^−1^ (H_CON240_/Q_CON240_); (b) the functional ratios at 30 °·s^−1^ (H_ECC30_/Q_CON30_) and 240 °·s^−1^ (H_ECC240_/Q_CON240_); and c) the mixed ratio (H_ECC30_/Q_CON240_). 

### 2.3. Electromyography Measurements

To monitor muscle activation of the hamstrings and the quadriceps, EMG measurements were performed in 33 participants. All EMG, torque and angular position signals were simultaneously recorded at 1000 Hz using a Biopac MP100 Acquisition Unit (Biopac Systems Inc., Goleta, California). Raw data were displayed on-line and they were analyzed using the Acknowledge software (Version 3.9.1., Biopac System Inc., Goleta, CA, USA). For EMG data recording, sets of bipolar bar surface electrodes with a distance between electrodes of 1 cm (TSD 150B, Biopac System Inc., Goleta, CA, USA) were used. The electrodes were placed on the rectus femoris (RF) and biceps femoris (BF) muscles. The skin area where the electrodes were placed was first shaved to remove any dead cells and it was then cleaned using alcohol wipes. To identify the precise electrode positions we scanned each muscle using an ultrasound system (SSD-3500, ALOKA, Japan) with a linear array probe of 6 cm length and a wave frequency of 7.5 MHz. Because the participant’s thigh in the seated position may exert pressure on the electrodes of the back thigh, we used a special chair attachment underneath the thigh, which was customized such that it allows empty space between the surface of skin and the dynamometer chair. 

**Figure 2:**
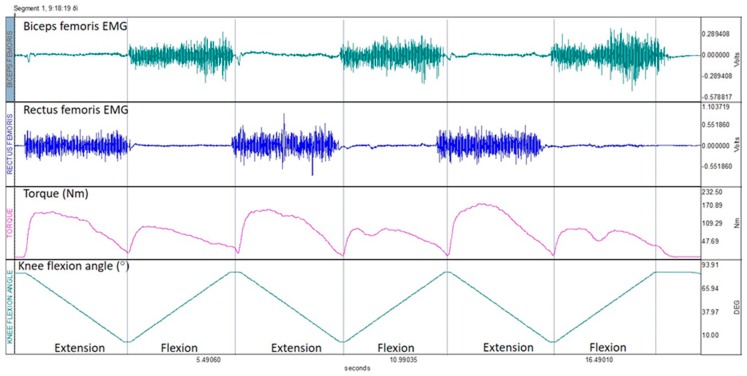
An example of raw EMG, torque and knee flexion angle data collected during three successive knee extension and flexion efforts from one participant. The data were obtained during a concentric test at 30 °·s^−1^.

The input impedance of the EMG signal was 10 MΩ, the common rejection ratio was 130 dB and the amplification gain was 1000 (Figure 2). A band pass filter, with a low cut-off frequency of 15 Hz and a high cut-off frequency of 450 Hz was applied to reduce noise that originated from surrounding sources of electricity and movement artifacts. The signal was then full-wave rectified and the root mean square (RMS) was calculated using a 10-sample sliding window. We then applied a 10-point moving average to smooth the signal to allow an easier identification of the peak RMS value from the EMG—time curves. 

The raw EMG signals obtained during the MVC efforts were subsequently used for normalizing the signals. Particularly, from the RMS—time curves obtained during the MVC trial, we took the average value for a 2-s period where the torque signal was least variable. Subsequently, all signals obtained during isokinetic tests were normalized against the isometric MVC values. For each testing condition, the peak normalized RMS signal during each isokinetic trial was used for further analysis. 

### 2.4. Reliability Testing and EMG Measurements

The participants who underwent EMG measurements returned one week after the first testing session for a retest, to determine the reliability of the measurements.

### 2.5. Statistical Analyses

An ICC was calculated to access reliability (ICC2,1) based on the peak torque per session. When the ICC ranged from 0.70 to 0.89 the reliability was considered as high while an ICC greater than 0.90 indicated very high reliability [32]. In addition, we estimated the standard error of measurement (SEM).

The data were first examined for normality using the Kolmogorov test. Subsequently, three-way analysis of variance (ANOVA) designs were used to examine the differences in conventional H:Q ratios between two types of muscle action (eccentric, concentric), three hip testing positions (H60, H90, H120) and two angular velocities (30, 240 °·s^−1^). Differences in functional H:Q ratios between hip flexion angles (90, 110°) at two angular velocities (30, 240 °·s^−1^) were checked using a two-way ANOVA. A one-way ANOVA was applied to establish the hip flexion angle influence on the mixed (H_ECC30_/Q_CON240_) ratio. Finally, the effects of hip angle, muscle action and angular velocity on torque values were tested using three-way ANOVA designs.

In case of significant interaction or main effects, we first applied a simple-effects and then Tukey’s post hoc tests to test which pair of means was significantly different. The level of significance was set at *p* < 0.05.

## 3. Results

### 3.1. Reliability Measurements

The results for the test-retest reliability of the H:Q ratios are presented in Table 1. The calculated ICC ranged from 0.86 to 0.93 for H:Q peak torque ratios and from 0.79 to 0.88 for normalized peak EMG measurements. The SEM ranged from 0.02 to 0.15 for the H:Q ratios and from 2.62% to 8.25% of MVC for the normalized EMG measurements.

### 3.2. H:Q Peak Torque Ratios

The results for all estimated H:Q ratios are presented in Table 2. The ANOVA indicated non-significant effects of hip flexion angle and speed on conventional ratios (*p* > 0.05). There was a statistically significant hip by angular velocity interaction (*p* < 0.05). Post-hoc Tukey tests indicated that the functional H:Q ratio 30 °·s^−1^ did not differ between the three hip testing positions (*p* > 0.05). In contrast, the same ratio at 240 °·s^−1^ was significantly greater at H90 compared with H60 and H120 positions (*p* > 0.05). Furthermore, there was a statistically significant increase of this ratio as angular velocity increased from 30 °·s^−1^ to 240 °·s^−1^ (*p* < 0.05). Finally, the mixed ratio was significantly greater at H90 compared to H60 and H120 testing positions (*p* < 0.05). 

### 3.3. Peak Torque

**Figure 3:**
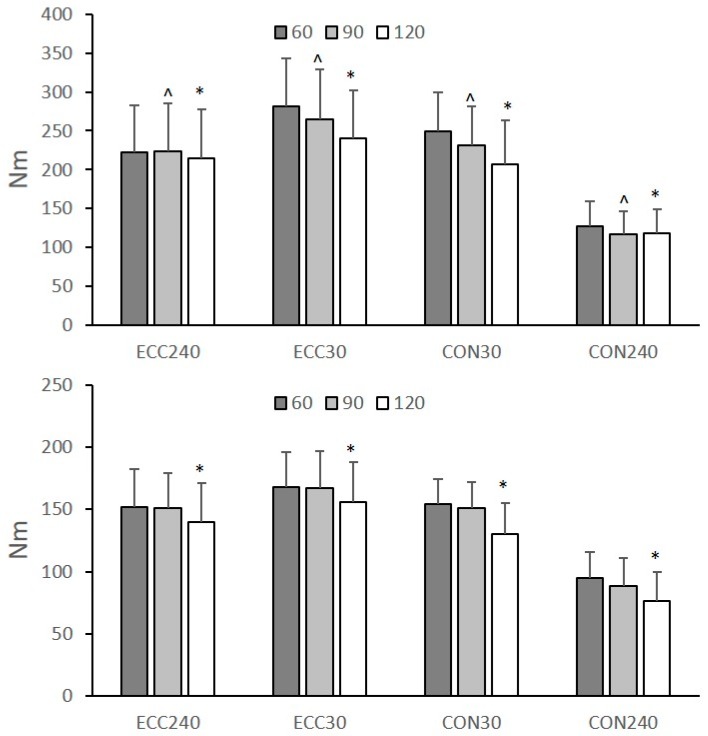
Mean group (N = 73) values of quadriceps and hamstrings torque values at hip flexion angles of 60°, 90°, and 120° during concentric (CON) and eccentric (ECC) tests at 30 °·s^−1^ and 240 °·s^−1^. Error bars indicate standard deviation (* significantly different compared with 60° and 90°, ^ significantly different compared with 60°, *p* < 0.05).

The peak torque at different hip angular positions, angular velocities and types of muscle action is presented in Figure 3. The ANOVA results showed a non-significant three-way interaction effect on peak Q and H torque (*p* > 0.05). The effect of hip flexion angle on peak Q and H torque was statistically significant (*p* > 0.05). Post-hoc Tukey tests showed that peak Q torque (collapsed across muscle action and angular velocities) significantly decreased with increasing hip flexion angle from H60 to H90 (*p* < 0.05) and from H90 to H120 (*p* < 0.05). Peak H torque was not significantly different between H60 and H90 tests, but it was significantly lower in H120 than H90 and H60 (*p* < 0.05). Peak torque of both muscles was greater during eccentric than concentric tests and decreased from 30 °·s^−1^ to 240 °·s^−1^ (*p* < 0.05).

### 3.4. Normalized EMG

Table 3 presents the normalized EMG values of the two muscles. There was a non-significant three-way interaction effect on peak Q and H normalized EMG (*p* > 0.05). The effect of hip flexion angle and angular velocity on normalized EMGs was non-statistically significant (*p* > 0.05). Normalized EMG values of both muscle groups were lower during eccentric than concentric tests (*p* < 0.05). 

## 4. Discussion

The main findings of this study are, first, that the hip flexion angle did not influence the conventional H:Q ratio, second, that the functional ratio at the faster angular velocity and the mixed H:Q ratio from the standard seated position (H90) were greater than the ratio measured at the other two hip flexion angles and, third, that no systematic effects of the hip flexion angle on the recorded muscle activation patterns were found. To our knowledge, no study has simultaneously examined the changes in peak H and Q torque between 60°, 90°, and 120° hip flexion angles.

Selecting a greater hip flexion angle for knee strength testing increases the length of the bi-articular hamstrings [33], it decreases the length of the bi-articular rectus femoris while the mono-articular muscles’ length is unaffected [26]. Consequently, one might expect that isokinetic testing from a greater muscle length (H120) would increase H peak torque and decrease Q torque. Our results showed that, amongst the three hip flexion angles, testing from a greater hip flexion angle changed almost equally the peak concentric torque of both muscle groups (Figure 3) and, hence, it had no effect on the conventional ratios (Table 1). This is in disagreement with previous studies which reported that an increase of the angle of hip flexion from 0° to 90° is accompanied by a greater change of peak H torque than the peak Q torque [23,25,34]. However, a detailed examination of the results reported by Guex et al. [23] indicates that when the hip flexion angle increases from 60° to 90°, then the rate of change of peak torque is similar for both muscle groups which is in line with present findings. Our results extend previous observations [23,25,34] as it appears that the peak concentric torque of both muscles decreased similarly when the test is performed from an even greater hip flexion angle (H120) than the standard seated position (H90) (Figure 3). This indicates that evaluation of muscle strength imbalances around the knee joint using isokinetic concentric tests is not dependent on the hip flexion angle.

The results showed that the hip flexion angle did not affect the functional ratio at 30 °·s^−1^ (Table 2). This agrees with Guex et al. [23] who found similar results for the functional H:Q ratio at 60 °·s^−1^. In contrast, the functional ratio at 240 °·s^−1^ was greater in H90 compared the other two testing positions (Table 2). This is in line with Deighan et al. [20] who found that hip flexion angle influenced the functional H:Q ratio at the faster angular velocity and not at the slower angular velocity. This is a result of a greater effect of hip flexion angle on the recorded H_ECC_ torque values at 240 °·s^−1^ than the corresponding changes in Q_CON_ torque at the same velocity (Figure 3). The reason for this selective effect of hip flexion angle on Hecc torque at the faster angular velocity is unclear. In theory, the hamstrings would be expected to exert the greatest torque at the most lengthened position, due to a greater utilization of the long tendons and aponeurosis (in-series elastic components) of the hamstrings. However, this was not the case, as H torque values were much greater from the H90 position than the most lengthened position (H120). It has been shown that when the hip flexion angle is 90° and the knee is extended more than 45°, the biceps femoris long head operates on the descending limb of the sarcomere force–length curve [33]. If this is the case, then further flexion of the hip may increase the length of hamstrings thus reducing their maximum force potential.

The mixed ratio (H_ECC30_/Q_CON240_) was greater in the H90 position compared with the other two positions (Table 2). There are no studies which have previously examined the influence of hip flexion angle on the mixed H:Q ratio. Nevertheless, the decrease of the mixed H:Q ratio at H120 is due to a greater decrease of the peak H_ECC_ at 30 °·s^−1^ compared with the other hip positions while peak Q_CON_ torque at 240 °·s^−1^ did not differ between the three positions (Figure 3). As with the functional H:Q ratio, this may indicate that the effect of hip flexion angle differs between eccentric and concentric tests and between quadricep and hamstring muscle torques. This is in line with Deighan et al. [20] who reported that the magnitude of hip flexion angle effect was significant for the Q_CON_ torque at fast angular velocity, Q_ECC_ torque at slow angular velocity and H_ECC_ torque at fast angular velocity. Guex et al. [23] reported that an increase of the hip flexion angle from 30° to 90° increased H_ECC_ peak torque at all speeds but it had almost no effect on Q_ECC_ torque at all speeds.

The peak RMS of the rectus femoris and biceps femoris was unaffected by the change in the testing position (Table 3). This is in agreement with previous studies on peak hamstring EMG activation [22,23,30] while Lunnen et al. [35] reported greater hamstrings EMG activity at a larger hip flexion angle. Furthermore, our results extend previous findings on minimal changes in the EMG of both muscle groups during isokinetic concentric tests between different hip angles [31]. The absence of muscle activity differences may be a combination of three factors: first, that given the short range of hip flexion angles examined (60°), the change in the length of each muscle-tendon unit was relatively small, second, that the rectus femoris is the only bi-articular muscle of the quadriceps muscle group and, hence, its activation may not have been significantly affected by short changes in hip flexion angle and, finally, that the observed changes in exerted torque at greater angles may not be due to alterations in neuromuscular activation but due to a different involvement of the passive components of the muscle-tendon unit [22]. 

The results have some implications for routine isokinetic testing of athletes, especially prior to the beginning of the competitive season. Several studies have suggested that there is no association between the H:Q ratio and hamstring injury in athletes from various sports [5,15,16,17]. However, recent evidence in soccer indicates that those players which display H:Q below a certain threshold value are more likely to sustain an injury [6]. Based on the significant increase in H:Q ratio from 0° to 90° of hip flexion, previous studies emphasized that selection of hip flexion angle is important for screening athletes for muscle strength imbalances [20,23]. Nevertheless, routine isokinetic assessment of peak muscle strength in soccer players is performed using the standard seated position [5,6,17,18]. Our results indicate that modification of the standard seated position by changing the hip angle by 30° does not influence the conventional H:Q ratio. In contrast, evaluation of the functional (only at fast speed) and the mixed H:Q ratios was different between the three angular positions. The selection of the hip flexion angle depends on the type of sport and the associated mechanisms that lead to injury. For sprinting hamstring injuries, Guex et al. [23] proposed that monitoring knee strength imbalance should be performed at 80° of hip flexion as it is more specific for the sprinting biomechanics. This sprint-type of hamstring injury is very common in soccer [2]. Our experimental design does not allow safe conclusions with regards to which hip flexion angle has better prediction capacity. When the aim of isokinetic evaluation is to monitor muscle strength imbalances at longer muscle lengths of the hamstrings after injury or following application of exercise programs at long muscle lengths [28,29], then the use of a greater hip flexion angle for routine strength testing may be particularly useful. Future research is necessary to verify if the isokinetic protocol from a different hip flexion angle has greater predictive ability than the traditional protocol. 

Future research can also examine the potential implications of changing hip flexion angle on functional tasks. For example, studies have commented that muscle co-activation plays a role of knee joint stability and it is a function of knee joint angle [21,36,37]. In this respect, small changes in hip flexion angle can selectively alter relative torque production of the two antagonistic muscle groups, resulting in changes in strength balance around the knee. This may be linked with the observation that an increase in hip flexion angle coupled with changes in knee flexion angle may alter the peak vertical and horizontal posterior ground reaction forces during landing [38]. 

## 5. Conclusions

For the range of hip flexion angles tested, routine isokinetic assessment of the conventional H:Q ratio and the functional H:Q ratio at slow speed can be performed at any of these three hip flexion angles. Should assessment of the fast-angular velocity functional H:Q ratio as well as the mixed ratio be required, then selection of hip flexion angle has an influence on the isokinetic assessment.

## Figures and Tables

**Table 1:** Intraclass correlation coefficients (ICCs) and standard error of measurement (SEM) resulting from comparison of test and retest values of H:Q torque ratios and normalized EMG values (N = 33; H = Hamstring; Q = Quadriceps, CON = Concentric, ECC = Eccentric, RF = Rectus femoris, BF = Biceps Femoris, 30 = 30 °·s^−1^, 240 = 240 °·s^−1^ )

Hip Angle (°)	60		90		120	
	ICC	SEM	ICC	SEM	ICC	SEM
	**H:Q Peak torque ratio**					
Conventional						
H_CON30_/Q_CON30_	0.93	0.04	0.92	0.03	0.90	0.02
H_CON240_/Q_CON240_	0.90	0.05	0.90	0.04	0.89	0.05
Functional						
H_ECC30_/Q_CONC30_	0.88	0.05	0.88	0.05	0.89	0.05
H_ECC240_/Q_CON240_	0.90	0.09	0.87	0.15	0.86	0.12
Mixed						
H_ECC30_/Q_CON240_	0.86	0.08	0.87	0.11	0.88	0.09
	**Normalized EMG**					
RF_CON30_	0.88	4.85	0.85	3.87	0.86	2.62
RF_CON240_	0.83	7.01	0.84	4.80	0.85	4.26
RF_ECC30_	0.86	6.73	0.89	4.31	0.88	4.50
RF_ECC240_	0.79	7.79	0.84	7.20	0.82	7.21
BF_CON30_	0.83	4.95	0.87	2.88	0.85	3.49
BF_CON240_	0.82	3.82	0.85	5.03	0.82	5.52
BF_ECC30_	0.87	5.05	0.86	5.61	0.84	6.00
BF_ECC240_	0.79	8.25	0.83	7.01	0.82	7.21

**Table 2:** Mean (±SD) group hamstrings to quadriceps peak torque ratios. The conventional ratios were calculated at concentric 30 °·s^−1^ (H_CON30_/Q_CON30_) and at 240 °·s^−1^ (H_CON240_/Q_CON240_). Similarly, the functional ratios at 30 °·s^−1^ (H_ECC30_/Q_CON30_) and 240 °·s^−1^ (H_ECC240_/Q_CON240_) and the mixed ratio as the H_ECC30_/Q_CON240_.

	Hip Flexion Angle (°)		
Ratio	60	90	120
Conventional			
H_CON30_/Q_CON30_	0.61 ± 0.09	0.62 ± 0.11	0.65 ± 0.15
H_CON240_/Q_CON240_	0.68 ± 0.12	0.69 ± 0.13	0.64 ± 0.16
Functional			
H_ECC30_/Q_CONC30_	0.70 ± 0.11	0.75 ± 0.14	0.78 ± 0.15
H_ECC240_/Q_CON240_	1.15 ± 0.24 ^	1.29 ± 0.35 ^*	1.17 ± 0.33 ^
Mixed			
H_ECC30_/Q_CON240_	1.23 ± 0.26	1.34 ± 0.35 *	1.24 ± 0.39

N = 73; * significantly different compared with hip flexion angle of 60° ^ significantly different compared with value at 30 °·s^−1^.

**Table 3:** Mean (±SD) normalized root mean squared EMG of the rectus femoris (VM) and biceps femoris (BF) at concentric (CON) and eccentric (ECC) angular velocity of 30 °·s^−1^ and 240 °·s^−1^ at three different hip testing positions.

	Hip Flexion Angle (°)		
Test	60	90	120
RF_CON30_	111.57 ± 20.26 *	104.77 ± 28.68 *	95.50 ± 18.40 *
RF_CON240_	123.02 ± 28.90 *	103.87 ± 31.03 *	107.65 ± 21.90 *
RF_ECC30_	100.84 ± 30.49	84.05 ± 26.20	86.48 ± 22.93
RF_ECC240_	100.94 ± 33.24	88.80 ± 25.96	95.76 ± 24.82
BF_CON30_	102.48 ± 31.86 *	108.51 ± 29.57 *	92.96 ± 20.58 *
BF_CON240_	95.79 ± 31.10 *	102.03 ± 24.10 *	106.59 ± 34.41 *
BF_ECC30_	84.66 ± 30.98	91.31 ± 28.64	86.94 ± 20.86
BF_ECC240_	86.66 ± 33.58	89.06 ± 27.63	83.45 ± 30.50

N = 33; * significantly different compared with eccentric value at *p* < 0.05.
